# Excellent Antibacterial Properties of Silver/Silica–Chitosan/Polyvinyl Alcohol Transparent Film

**DOI:** 10.3390/ijms25158125

**Published:** 2024-07-25

**Authors:** Taoyang Cai, Shangjie Ge-Zhang, Chang Zhang, Pingxuan Mu, Jingang Cui

**Affiliations:** 1 College of Science, Northeast Forestry University, Harbin 150040, China; cty@nefu.edu.cn (T.C.);; 2 Aulin College, Northeast Forestry University, Harbin 150040, China

**Keywords:** transparent film, chitosan (CS), polyvinyl alcohol (PVA), antibacterial property

## Abstract

Transparent films with excellent antibacterial properties and strong mechanical properties are highly sought after in packaging applications. In this study, Ag/SiO_2_ nanoparticles were introduced into a mixed solution of chitosan (CS) and polyvinyl alcohol (PVA) and a Ag/SiO_2_-CS-PVA transparent film was developed. The excellent properties of the film were confirmed by light transmittance, water contact angle tests and tensile tests. In addition, for the antibacterial test, the antibacterial properties of the sample against Gram-negative bacteria (*Escherichia coli*) and Gram-positive bacteria (*Staphylococcus aureus*) were explored, and the average size of the bacteriostatic circle was measured by the cross method. The final results show that Ag/SiO_2_-CS-PVA transparent film has the advantages of good antibacterial properties, high transparency and high mechanical strength.

## 1. Introduction

Packaging plays a vital role in maintaining food quality and safety, prolonging shelf life and minimizing pollution risk [1,2,3,4]. Among all kinds of packaging materials, transparent film has attracted considerable attention because it can keep packaged products fresh and provide visibility at the same time [5,6]. However, a significant disadvantage of traditional transparent film is its limited antibacterial performance, which will affect food safety and shelf life [7]. In addition, due to the theory of sustainable development, researchers have shown a growing interest in developing biodegradable transparent polymer films to combat white pollution [8,9].

Chitosan (CS) is a natural biodegradable polymer, which is derived from the exoskeleton of crustaceans, fungi and insects. It has good biocompatibility and biodegradability, and can be used as a protective layer in packaging materials. In addition, CS also has certain antibacterial properties, which helps to protect food from bacterial pollution [10,11]. However, its solubility in various solvents is poor, as it will form a gel in neutral and acidic solutions, and its mechanical properties are also poor [12]. Polyvinyl alcohol (PVA) is a synthetic water-soluble polymer with good chemical resistance, sealing performance and mechanical properties, which has been used in many biomaterials [13,14]. Generally speaking, PVA is usually used as a sealing layer, which can effectively block the penetration of gas and moisture and maintain the freshness of food [15]. Mixing these two polymers can significantly improve the solubility of chitosan in various solvents and improve the mechanical properties of the obtained composites [16,17]. Moreover, the hydroxyl group on the PVA molecule and the hydroxyl group or amino group on the CS molecule generate hydrogen bonds, which can be more closely mixed [18]. Researchers have improved the properties of the film by adjusting the ratio and structure of CS-PVA, such as its transparency, antibacterial properties and barrier properties [19].

Nanoparticles are increasingly introduced into polymer materials to improve the mechanical properties of films, and some nanoparticles can also give additional antibacterial properties [20]. In this case, the integration of Ag nanoparticles (Ag NPs) into a polymer matrix has become a promising method to endow transparent films with antibacterial properties. Ag shows strong antibacterial activity by destroying the microbial cell membrane and interfering with important cell functions, making it an ideal candidate material for food packaging applications [21,22]. The existence of a large number of silanol groups in the nano-silica (SiO_2_ NP) structure helps to form strong hydrogen bonds, which leads to a closer interface between the two polymers in the composite system [23]. Nano-silica, as a carrier, can improve the stability of Ag nanoparticles and prolong their life in applications [24]. In addition, the addition of SiO_2_ to the thin film can enhance its physical properties, such as strength and stability [25].

In this study, we propose a new transparent film, which consists of a mixture of chitosan (CS) and polyvinyl alcohol (PVA) and silver nanoparticles encapsulated in silica nanoparticles (Ag/SiO_2_). The film provides a unique combination of transparency, mechanical strength and antibacterial properties, making it very suitable for ensuring the safety and quality of packaging. In the following sections of this paper, the experimental methods, results and discussions will be deeply studied to fully understand the performance and potential application of this innovative transparent film.

## 2. Results and Discussion

### 2.1. Optical Properties

**Figure 1:**
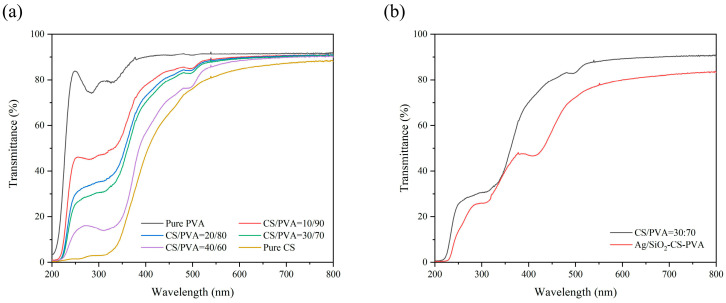
Transmittance from 200 nm to 800 nm of CS/PVA composite films at different volume ratios (**a**) and before/after adding Ag/SiO_2_ nanoparticles (**b**).

Good visibility of packaging film is necessary, as it can enable consumers to intuitively evaluate the freshness of products and enhance their attractiveness and recognition [26]. Therefore, the transparency of the film in terms of visibility must be considered. Figure 1a reports the transparency of CS/PVA-blend films at different proportions. The pure PVA film has the highest transparency, and the transmittance is between 90.7% (at 450 nm) and 91.2% (at 800 nm). With an increase in CS, the value of transparency decreases, and the value of the pure CS film is the lowest, and the transmittance from 450 nm to 800 nm is between 65.3% and 88.0%. When the volume ratio of CS/PVA is 10:90, less CS barely weakens the light transmittance. The transmittance at 800 nm reached 90.5%, almost the same as that of the pure PVA film, and the CS/PVA = 10:90 firm reached 84.0% at 450 nm. The light transmittance of CS/PVA films with volume ratios of 20:80 and 30:70 decreased slightly to 81.4–90.3% and 80.0–89.8% (from 450 nm to 800 nm), respectively. Further increasing the proportion of CS, when the volume ratio of CS/PVA reaches 40:60, the light transmittance of the film decreases noticeably, and it is only 71.5% at 450 nm. The obvious yellow appearance may affect consumers’ accurate evaluation of the product. When the volume ratio of CS/PVA is 30:70, the light transmittance before and after adding Ag/SiO_2_ nanoparticles is shown in Figure 1b. After adding the nanoparticles, the light transmittance at 800 nm decreased to 83.1%, and at 450 nm, it decreased to 57.8%. This is because Ag/SiO_2_ nanoparticles have a certain scattering ability, and the nanoparticles in the film introduce additional light-scattering centers, which leads to multiple scatterings of light when passing through the film, thus reducing the intensity of the transmitted light [27]. In addition, the increase in surface roughness will also enhance diffuse reflection and decrease transmittance [28]. The transmittance of Ag/SiO_2_-CS-PVA is still more than 77% at 550 nm, the most sensitive for human eyes [29].

### 2.2. Mechanical Properties

In the food packaging industry, the key role of food packaging film is to ensure its integrity, which gives it sufficient strength and durability to cope with various pressures and challenges arising in the process of transportation, handling and storage [30]. The mechanical properties of CS/PVA-blend films with different proportions are shown in Table 1. With an increase in the CS volume ratio from 10% to 100%, the film showed a continuous increase in tensile strength (from 14.6 MPa to 28.4 MPa), but the elongation at break rapidly decreased from 217.5% to 28.2%. The addition of more CS with higher rigidity and strength and the interaction of hydrogen bonds between CS and PVA improved the tensile strength a little, but it also limited the segment slip and the adjustment between the molecular chains of PVA during stretching, resulting in a decrease in deformability, thus reducing the elongation at break. After adding 3% Ag/SiO_2_ nanoparticles to the CS:PVA = 30:70 group, the tensile strength increased to 25.5 MPa, which was caused by the high rigidity and specific surface area of the nanoparticles, and the uniformly distributed nanoparticles provided an effective stress transfer [31].

### 2.3. Micromorphology

**Figure 2:**
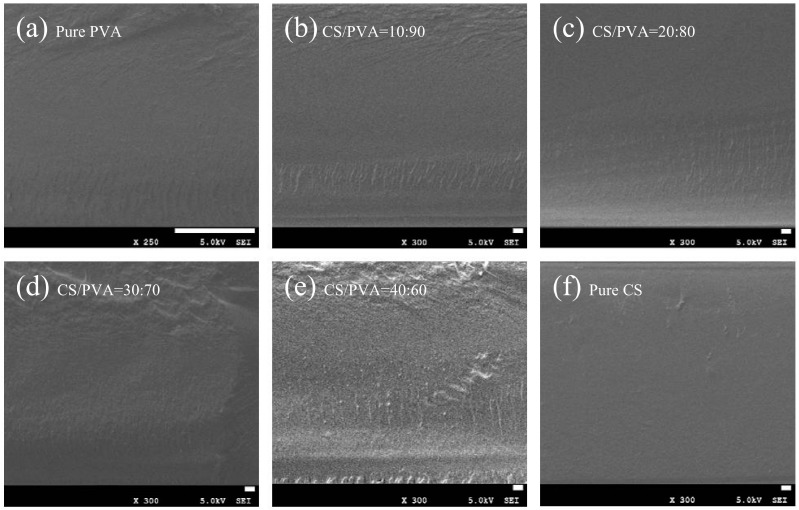
SEM images of composite membranes at different volume ratios of CS/PVA. (**a**) Pure PVA, (**b**) CS/PVA = 10:90, (**c**) CS/PVA = 20:80, (**d**) CS/PVA = 30:70, (**e**) CS/PVA = 40:60, (**f**) pure CS.

Figure 2 shows SEM images of cross-sections of the CS/PVA-blend films at different proportions. Different from pure PVA and pure CS films, with a continuous and uniform morphology (Figure 2a,f), the broken surface of CS/PVA films is rougher. For the film blend with 10–30% CS by volume (Figure 2b–d), the compatibility between them was good, and no obvious agglomeration was observed. In addition, no irregularities such as pores and cracks were detected. However, for the CS/PVA = 40:60 group (Figure 2e), the film had obvious cross-section roughness and adhesion at the same time, and its compatibility was poor. The EDS energy spectrum in Figure S1 shows that Ag was successfully introduced into SiO_2_ nanoparticles and distributed uniformly [32].

### 2.4. FTIR

**Figure 3:**
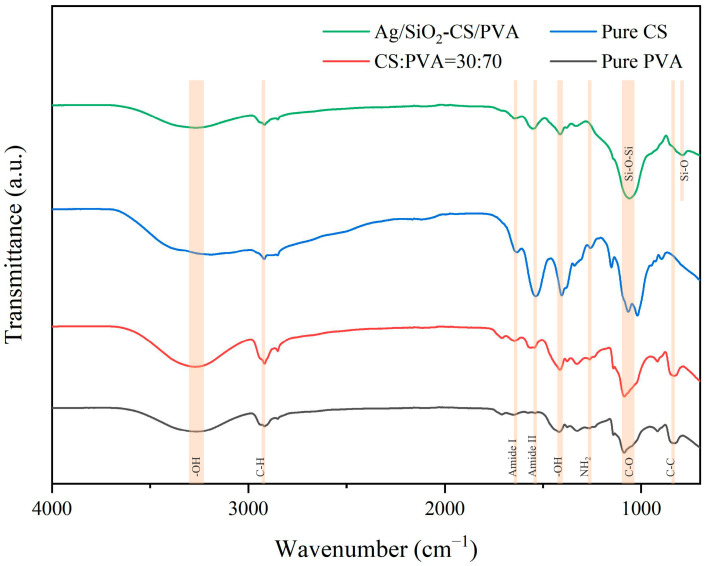
FTIR spectra of CS/PVA films at different volume mixing ratios.

Considering the compatibility, optical properties and mechanical properties of the film, the group CS/PVA = 30:70 was selected to dope Ag/SiO_2_ nanoparticles, which was then called Ag/SiO_2_-CS/PVA film. As shown in Figure 3, the broad peak of the pure PVA film at 3280 cm^−1^ comes from the tensile vibration of the -OH group, while at 2918 cm^−1^, it comes from the tensile vibration of C-H. The bending vibration of OH produces a peak at 1418 cm^−1^, and the peaks at 1090 cm^−1^ and 836 cm^−1^ correspond to the tensile vibrations of C-O and C-C, respectively. Different from the PVA film, there are obvious absorption peaks of amide I and amide II in the pure CS film at about 1640 cm^−1^ and 1535 cm^−1^, and these peaks are obvious with an increase in the CS content, which are formed by the deacetylation part in CS [33]. The peaks at 1152 cm^−1^ and 899 cm^−1^ are thought to be caused by their saccharine structure [34]. The amino characteristic peak at 1254 cm^−1^ is weak and can only be observed in pure CS films. The peak at 1070 cm^−1^ belongs to the C-O tensile vibration of CS, but it finally moves to 1090 cm^−1^ with a decrease in the CS volume ratio [35]. In addition, the peak at 836 cm^−1^ of the pure PVA film gradually disappears with an increase in the CS volume ratio. PVA and CS have good compatibility. The molecular structure of PVA contains a large number of hydroxyl (-OH) groups, which can form hydrogen bonds with many amino (-NH_2_) and hydroxyl (-OH) groups rich in CS, thus attracting and being compatible with each other [36]. A band near 784 cm^−1^ of Si-O bending vibration and an antisymmetric stretching vibration peak of Si-O-Si at 1063 cm^−1^ were observed, which indicated the successful introduction of Ag/SiO_2_ nanoparticles.

### 2.5. XRD

**Figure 4:**
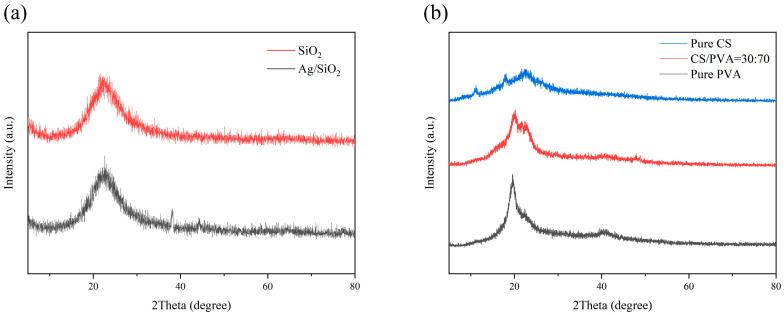
The XRD patterns of SiO_2_ nanoparticles before/after the introduction of Ag (**a**) and CS/PVA composite films (**b**).

The XRD images of SiO_2_ and Ag/SiO_2_ nanoparticles are shown in Figure 4a. Pure silica particles have only one diffraction peak (2θ = 22.8°), which is the characteristic diffraction peak of amorphous silica. For Ag/SiO_2_, there are four sharp diffraction peaks at 38.1°, 44.3°, 64.6° and 77.4°, corresponding to the (111), (200), (220) and (311) planes of silver, and elemental silver grows on SiO_2_. In Figure 4b, the XRD pattern of the pure CS films shows three low-intensity peaks, which are about 2θ = 11.4°, 2θ = 18.0° and 2θ = 22.5°, respectively. The pure PVA film has two peaks near 2θ = 19.6° and 2θ = 40.6°. The XRD pattern of the CS/PVA = 30:70 group after mechanical mixing shows a simple mixed pattern; that is, 2θ = 20.1° corresponds to the peak of the pure PVA film and 2θ = 22.8° corresponds to the peak of the pure CS film, indicating that there is no strong chemical reaction between them [35].

### 2.6. Antimicrobial Activity

As the representative strains of Gram-negative bacteria and Gram-positive bacteria, *Escherichia coli* and *Staphylococcus aureus* can reflect well whether a sample has an inhibitory effect on broad-spectrum bacteria. Because the bacteriostatic circles produced by the samples may be irregular, the average size of the bacteriostatic circles, measured by the cross method, can better evaluate the antibacterial ability of the sample against *Escherichia coli* and *Staphylococcus aureus*.

**Figure 5:**
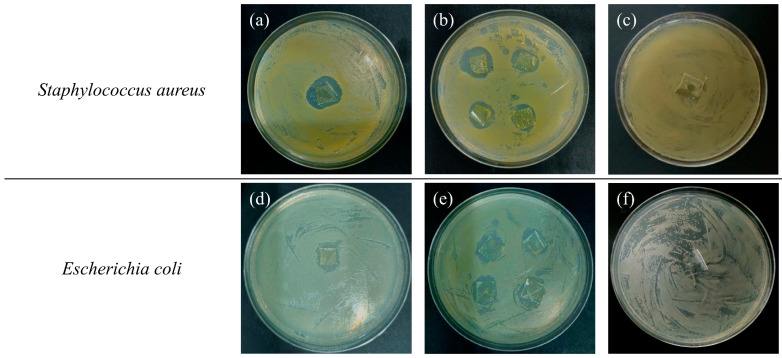
Antibacterial ability of Ag/SiO_2_-CS/PVA films (**a**,**b**,**d**,**e**) and pure PVA films (**c**,**f**).

As shown in Figure 5, *Staphylococcus aureus* colonies can produce fat-soluble, golden-yellow pigment, so the pigment is confined to the colonies and the colonies are yellow, in general, and smooth in surface (Figure 5c). *Escherichia coli* colonies are generally milky-white or yellowish (Figure 5f). For *Staphylococcus aureus*, Figure 5a,b show that only slight differences are found in the size of the bacteriostatic circles of four repeated experiments in Petri dishes, which shows the antibacterial stability of the film, and the average value of the bacteriostatic circle can also be measured. The antibacterial stability of *Escherichia coli* is illustrated in Figure 5d,e. Compared to the control culture, that is, the pure PVA film (Figure 5c,f), the Ag/SiO_2_-CS/PVA film formed clean and transparent areas, with average diameters of 17.5 mm (Figure 5a,b) and 14.0 mm (Figure 5d,e) in the *Staphylococcus aureus* colonies and *Escherichia coli* colonies, respectively, and the number of viable bacteria was significantly reduced.

Choosing silicon dioxide as a carrier and embedding Ag NPs into PVA/CS basement membranes can control the slow and continuous release of Ag^+^, which has a strong antibacterial effect on broad-spectrum bacteria [37]. After the contact reaction between Ag ions and bacteria, a small amount of Ag ions penetrates the cell wall to reach the cell membrane of microorganisms and adsorb with each other, damaging the inherent components of the microorganisms, leading to the cracking and death of the bacteria [38]. In addition, the Ag ions entering the cell can inhibit the enzyme activities related to maintaining the normal metabolic activities of bacteria [39]. When the bacteria lose their activity, metal ions will be released from the bacteria again, and sterilization activities will be repeated, so that the antibacterial effect is lasting. In addition, previous studies have shown that PVA/CS substrates also have a certain antibacterial ability [40].

### 2.7. Thermogravimetric Properties

**Figure 6:**
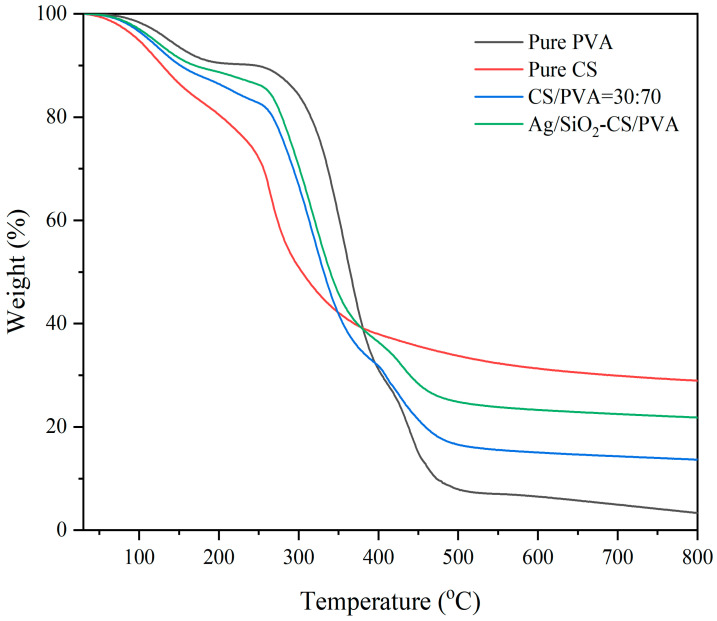
A typical TGA thermogram of pure PVA, pure CS, CS/PVA = 30:70 and Ag/SiO_2_-CS-PVA.

The thermal degradation of the samples at 30–800 °C was analyzed, and the results are shown in Figure 6. Pure PVA shows weight loss in three temperature ranges. First of all, the loss of physical water occurs from room temperature to 150 °C, which is a gentle downward trend (mass loss ~8%). Severe mass loss occurred between 270 °C and 385 °C, and the mass loss at this stage was as high as 55%, due to dehydration caused by the elimination of the hydroxyl side groups. The mass loss from 400 °C to 470 °C corresponds to the break in the PVA main chain, and the final residue is ~5% [41]. The thermal degradation of CS is carried out in two steps. The weight-loss process before 150 °C is considered to be caused by the loss of bound water and the crystallization of water contained in the material, as well as the loss of residual acetic acid when dissolving chitosan. At 250–300 °C, the thermal degradation rate was extremely high. At 300–700 °C, the degradation tended to be slow. Thermal degradation basically ended at 700 °C, and the final residual mass was as high as 29%. For the film sample of CS/PVA = 30:70, it shows the thermogravimetric results of the combination of pure PVA and pure CS, and the final residual mass is also between the thermogravimetric loss rates of pure PVA and pure CS. The introduction of Ag/SiO_2_ nanoparticles enhanced the thermal stability of the films, and Ag/SiO_2_-CS-PVA showed a thermogravimetric curve similar to CS/PVA = 30:70. However, the thermogravimetric residual mass of the films with Ag/SiO_2_ nanoparticles was higher than that of the films without particles at any temperature.

### 2.8. Water Contact Angle

**Figure 7:**
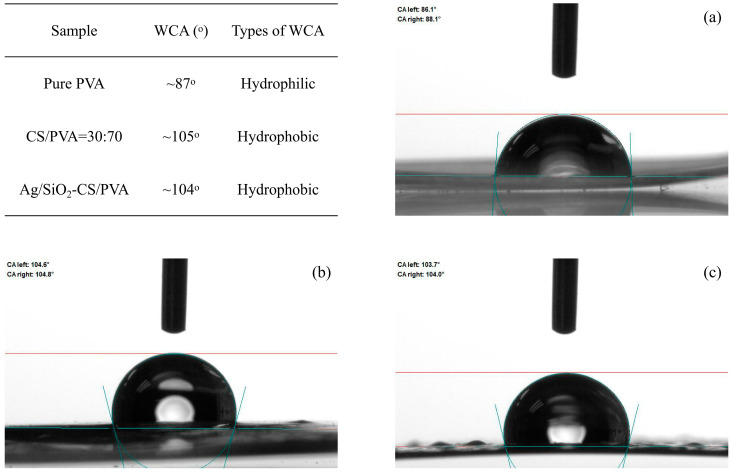
Static water contact angle of pure PVA surface (**a**), CS/PVA = 30:70 surface (**b**) and Ag/SiO_2_-CS-PVA surface (**c**).

The contact angle of water was evaluated by static wetting behavior, and the amount of droplets used in each evaluation was 5 μL. After standing until the hydrophobic angle remained unchanged, pictures were taken. As shown in Figure 7, the pure PVA film shows hydrophilic characteristics, and the water contact angle is ~87°. This is because PVA molecules contain a large number of hydroxyl groups (-OH), which can form hydrogen bonds with water molecules, giving PVA hydrophilicity. However, the films with CS/PVA = 30:70 and Ag/SiO_2_-CS-PVA both showed hydrophobic angles greater than 90°. The improvement in hydrophobicity was mainly attributed to the introduction of chitosan, which destroyed the hydrogen bond network inside PVA. The formation of hydrogen bonds between chitosan and PVA reduced the number of free hydroxyl groups and the interaction with water. In addition, the mixing of chitosan and PVA slightly enhanced the micro-roughness of the films, thus increasing the hydrophobicity of the whole film. In subsequent experiments, it will be necessary to modify the surface of the film with low-surface-energy substances, which may further improve the hydrophobicity of the film [42,43].

## 3. Materials and Methods

### 3.1. Materials

Nano-silica (SiO_2_ NPs, 30 nm), silver nitrate (AgNO_3_, 99.8%, AR), chitosan (CS, 200–400 mPa·s), polyvinyl alcohol (PVA-1799, alcoholysis degree of 98–99%), aminopropyl triethoxysilane (APTES, 99%, AR), acetic acid (CH_3_COOH, ≥99.8%, AR) and anhydrous ethanol (C_3_H_6_O, ≥99.8%, AR) were purchased from Shanghai Aladdin Biochemical Technology Co., Ltd. (Shanghai, China). *Escherichia coli*/*E.coli* (strain number: ATCC 25922) and *Staphylococcus aureus*/*S. aureus* (strain number: ATCC 6538) were purchased from Beijing Biological Conservation Center. All chemicals were used as received without further purification.

### 3.2. Preparation of Ag/SiO_2_ Nanoparticles

The preparation of Ag/SiO_2_ refers to the methods of Chi et al. and has been modified appropriately [44]. The volume ratio of 3-aminopropyl triethoxysilane (modifier) and anhydrous ethanol (modifying solvent) was 0.8:20 to prepare modified solution. Subsequently, 1 g of SiO_2_ was added to the modified solution, and the modified SiO_2_ was obtained by slightly stirring at 40 °C for 24 h. After ethanol cleaning, it was put into anhydrous ethanol solution containing 0.01 mol L^−1^ silver nitrate, and after 48 h of stirring at 40 °C in the dark, the modification of silica nanoparticles by silver ions was realized. Ag/SiO_2_ nanoparticles were prepared by washing them with deionized water and completely drying in an oven at 60 °C.

### 3.3. Preparation of CS-PVA Film

An amount of 2 g of CS was dissolved in 100 mL of 2% (*v*/*v*) acetic acid–deionized water solution and stirred at 60 °C for 1 h until it was completely dissolved to obtain a viscous and uniform CS solution. An amount of 3 g of PVA was dissolved in 100 mL of deionized water and stirred at 60 °C until it was completely dissolved to obtain a clear PVA aqueous solution. Subsequently, CS and PVA solutions were mixed according to different volume ratios (mixing volume ratios were 40:60, 30:70, 20:80, and 10:90), and 100 mL of blending solution was obtained. After ultrasonic treatment, they were poured into a 200 mm × 200 mm glassware and dried in an oven at 50 °C to form a film. After uncovering the film, they were left at room temperature for 24 h before use.

### 3.4. Preparation of Ag/SiO_2_-CS-PVA Film

Referring to the research results of Chen et al. [31], 3 wt% Ag/SiO_2_ nanoparticles were mixed into the CS-PVA blend, stirred at 60 °C for 30 min to form a uniform solution, and then ultrasonically treated and cooled to room temperature. The subsequent steps are the same as the preparation of the CS/PVA film.

### 3.5. Characterization

A cold-field emission scanning electron microscope (FE-SEM, JSM-7500F, JEOL Ltd., Akishima, Japan) was used to observe the micromorphology of the sample, and the accelerating voltage was 5.0 kV. When measuring the film, it was necessary to freeze it in liquid nitrogen in advance to fracture it, and a cooling energy spectrometer (EDS, OxfordX-Max, Oxford Instruments plc, Oxford, UK) connected to it was used to observe the elemental composition of the sample. An X-ray diffractometer (XRD, XRD-6100, Shimadzu Corporation, Kyoto, Japan) was used to measure the crystallinity of the sample; the scanning range was 5–85°, and the scanning rate was 8° min^−1^. A Fourier infrared spectrometer (FTIR, NicoletiS50, Thermo Fisher Scientific Inc., Waltham, MA, USA) was used to measure the chemical structure of the sample with a resolution of 4 cm^−1^ in the wave number range of 400–4000 cm^−1^. The mechanical properties of the samples were measured by a universal testing machine (UTM, Sanshi CMT-6305, MTS Systems Corporation, Shenzhen, China), including tensile strength and elongation at break. The transmittance of the sample was characterized by a UV-Vis spectrophotometer (UV-Vis, LAMBDA 1050+, PerkinElmer Inc., Waltham, MA, USA), and the testing range was 200–800 nm. The mass loss of the sample at 30 °C to 800 °C was measured by a simultaneous thermal analyzer (STA, Perkin Elmer STA 6000, PerkinElmer Inc., Waltham, MA, USA), and the heating rate was 10 °C min^−1^. The static water contact angles of different films were measured with a droplet volume of 5 μL, and the instrument used was a droplet shape analyzer (DSA100, Krüss, Krüss Scientific Instruments Inc., Hamburg, Germany). The agar diffusion method was used to evaluate the antibacterial activity of the film against *Escherichia coli* type 3 (Gram-negative) and *Staphylococcus aureus* type S33R (Gram-positive). The sample was inoculated with 10^7^–10^8^ CFU mL^−1^ bacterial suspension on a nutrient agar plate and the film (diameter 10 mm) was placed on the surface. After 24 h of culture at 37 °C, the diameter (mm) of the transparent zone was measured to determine the bacterial inhibition [25]. For the bacteriostatic experiment, the plate contact culture method was used to test the antibacterial ability of the sample materials against two common strains, *Escherichia coli* and *Staphylococcus aureus*, and it was evaluated by their bacteriostatic circles (mm). Specifically, the monoclonal colonies of the two strains were selected and dissolved in LB liquid culture medium (lysogeny broth) and sterilized by high-pressure steam for 20 min. The obtained bacterial liquid was incubated on a shaker at 37 °C and 200 rpm for 5 h until the bacteria grew to logarithmic phase (OD600 is about 0.6). The activated bacterial liquid was coated on a Petri dish (diameter 10 cm) containing LB solid medium (20 mL) by the coating plate method, and dry antibacterial materials of the same size were placed at an appropriate distance. Finally, the plate was placed in a constant-temperature incubator at 37 °C for 24 h. The results obtained are the average values of repeated plates.

## 4. Conclusions

In general, this study successfully prepared Ag/SiO_2_-CS-PVA transparent films by doping Ag-loaded SiO_2_ nanoparticles into a chitosan (CS)–polyvinyl alcohol (PVA) mixed solution. The films show good light transmittance (more than 77% at 550 nm), mechanical properties (tensile strength = 25.5 MPa), and certain hydrophobic properties (a static water contact angle of ~104°). The combination of silver ions released by Ag/SiO_2_ nanoparticles and CS jointly achieved an effect on Gram-negative bacteria (*Escherichia coli*) and Gram-positive bacteria (*Staphylococcus aureus*). Considering the cost of silver nanoparticles, future research may explore the use of plant-derived natural antimicrobial ingredients to impart antimicrobial properties to the film. In addition, we will further study the best ratio of Ag/SiO_2_ and CS/PVA. By doping Ag/SiO_2_ with different weight percentages into CS/PVA with different volume ratios, this orthogonal experiment is helpful to find the best balance among the various properties of the film.

## Figures and Tables

**Table 1:** Mechanical properties of CS/PVA films.

Sample	Tensile Strength (MPa)	Elongation at Break (%)
Pure PVA	14.6	217.5
CS:PVA = 10:90	16.3	173.4
CS:PVA = 20:80	18.8	128.3
CS:PVA = 30:70	20.9	112.6
CS:PVA = 40:60	22.1	88.7
Pure CS	28.4	28.2
Ag/SiO_2_-CS/PVA	25.5	76.7

## Data Availability

The original contributions presented in this study are included in the article/Supplementary Materials; further inquiries can be directed to the corresponding authors.

## Supplementary Materials

The following supporting information can be downloaded at https://www.mdpi.com/article/10.3390/ijms25158125/s1.
