# Supplementary figures and images for: Excellent Antibacterial Properties of Silver/Silica–Chitosan/Polyvinyl Alcohol Transparent Film
Source: Int J Mol Sci. 2024 Jul 25;25(15):8125. doi: 10.3390/ijms25158125 (PMC11311888; doi:10.3390/ijms25158125)

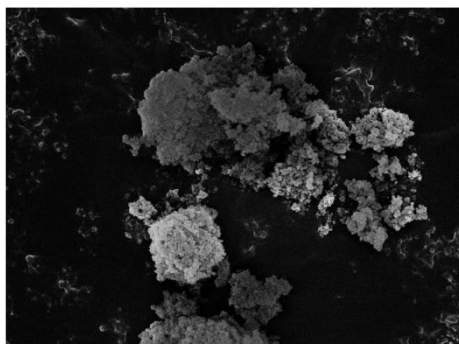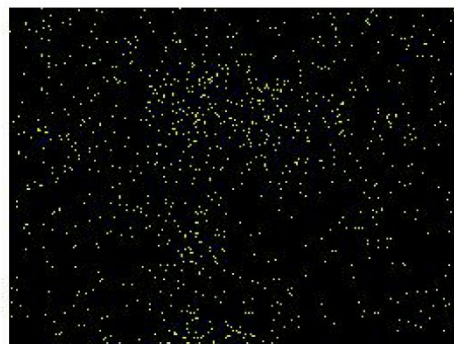

O Ka1

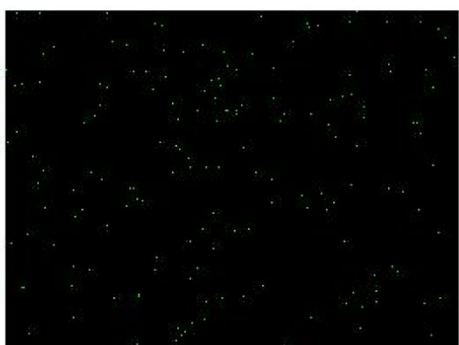

Ag La1

| Element | Atomic Percentage % |
|---------|---------------------|
| Si K    | 46.36%              |
| O K     | 50.71%              |
| Ag K    | 2.93%               |

Figure S1. EDS image of Ag/SiO<sub>2</sub> nanoparticles

Supplement: Supplementary file 1 [file ijms-25-08125-s001.zip › ijms-3118482-supplementary.pdf]
